# Activation of Adrenal Steroidogenesis and an Improvement of Mood Balance in Postmenopausal Females after Spa Treatment Based on Physical Activity

**DOI:** 10.3390/ijms20153687

**Published:** 2019-07-27

**Authors:** Pavla Honců, Martin Hill, Marie Bičíková, Dobroslava Jandová, Marta Velíková, Jiří Kajzar, Lucie Kolátorová, Jiří Bešťák, Ludmila Máčová, Radmila Kancheva, Milada Krejčí, Jaroslav Novotný, Ľuboslav Stárka

**Affiliations:** 1 Department of Rehabilitation Medicine, 3rd Faculty of Medicine, Charles University, 12808 Prague, Czech Republic; 2 Institute of Endocrinology, 11694 Prague, Czech Republic; 3 College of Physical Education and Sport Palestra, 19700 Prague, Czech Republic; 4 Priessnitz Spa Resort Jeseník, 79003 Jeseník, Czech Republic

**Keywords:** postmenopausal females, mood balance, spa treatment, adrenal, steroid metabolome

## Abstract

Spa treatment can effectively reestablish mood balance in patients with psychiatric disorders. In light of the adrenal gland’s role as a crossroad of psychosomatic medicine, this study evaluated changes in 88 circulating steroids and their relationships with a consolidation of somatic, psychosomatic and psychiatric components from a modified N-5 neurotic questionnaire in 46 postmenopausal 50+ women with anxiety-depressive complaints. The patients underwent a standardized one-month intervention therapy with physical activity and an optimized daily regimen in a spa in the Czech Republic. All participants were on medication with selective serotonin reuptake inhibitors. An increase of adrenal steroidogenesis after intervention indicated a reinstatement of the hypothalamic-pituitary-adrenal axis. The increases of many of these steroids were likely beneficial to patients, including immunoprotective adrenal androgens and their metabolites, neuroactive steroids that stimulate mental activity but protect from excitotoxicity, steroids that suppress pain perception and fear, steroids that consolidate insulin secretion, and steroids that improve xenobiotic clearance. The positive associations between the initial values of neurotic symptoms and their declines after the intervention, as well as between initial adrenal activity and the decline of neurotic symptoms, indicate that neurotic impairment may be alleviated by such therapy provided that the initial adrenal activity is not seriously disrupted.

## 1. Introduction

Almost 70 years ago, Mc Rae suggested that the adrenal gland is a crossroad of psychosomatic medicine [1]. A central concept of psychosomatic medicine is the close interconnection of mentality with body function and condition [2,3]. Numerous psychiatric disorders are associated with the overproduction of corticotropin-releasing hormone (CRH), a peptide hormone controlling the activity of the hypothalamic-pituitary-adrenal axis (HPAA) and regulating the synthesis of adrenal steroids. HPAA is commonly dysregulated in eating disorders, affective psychosis, addiction, Alzheimer’s and Parkinson’s diseases and progressive supranuclear palsy [4,5]. However, CRH overproduction in the central nervous system (CNS) and periphery and disruption of the HPAA are also typical for such somatic complaints as autoimmune, allergic, gastrointestinal, and chronic inflammatory diseases, as well as altered pain perception. In these diseases, the CRH operates on both peripheral and central levels [4,5]. The CRH indirectly operates in an anti-inflammatory fashion via the stimulation of anti-inflammatory cortisol and immunoprotective adrenal androgens [6], while the autocrine/paracrine peripheral effect is the opposite [7].

There is a well-known simulating effect of CRH on adrenocorticotropic hormone (ACTH) production and the subsequent ACTH-induced activation of glucocorticoid production in the *zona fasciculata* (ZF). This peptide hormone acts as a potent adrenal androgen secretagogue acting in the *zona reticularis* (ZR). While CRH alone does not influence the synthesis of pregnane steroids in the ZF, it does induce rapid synthesis of adrenal androgens in the ZR [8]. The primary adrenal androgens dehydroepiandrosterone (DHEAS) and its sulfate (DHEAS) play an important role in the maintenance of immunity, musculoskeletal integrity and cardiovascular health, and their levels decrease to less than 20% of their maximal values during life, with parallel cell shrinkage in the ZR [9].

An amplified activation of CRH has been observed in Alzheimer’s disease, and an even stronger increase has been observed in major depression. The effect of CRH on ACTH release is strongly potentiated by vasopressin. This hormone shows a parallel increase with CRH during the chronic activation of hypothalamic paraventricular neurons and other sources in the CNS. While vasopressin further promotes ACTH release in humans, oxytocin, known as the love hormone, inhibits it. Additionally, a close interaction has been reported with stimulated HPAA activity leading to a suppressed functioning of the hypothalamic-pituitary-gonadal-axis in order to save energy [10,11]. Apart from the intra-adrenal CRH receptor/ACTH system, the type 1 receptors for CRH, which are widely expressed in the ZR, may directly mediate the synthesis of adrenal androgens [12].

While the ZR is known as a successor of the fetal adrenal zone (FZ) in anthropoid primates, the primate FZ may serve as the most appropriate model for investigating relationships between CRH and adrenal androgens. These associations have been more thoroughly studied. The data from the FZ as reported by Siriani et al. [13] showed increased mRNA expression of the genes needed for DHEAS production, such as steroidogenic acute regulatory protein, cholesterol desmolase (CYP11A1), steroid C17-hydroxylase-C17,20-lyase (CYP17A1), and type 2A1 steroid sulfotransferase (SULT2A1), as well as the upregulation of type 1 CRH receptors after 24 h treatment of FZ cells with CRH, CRH-related peptide urocortin or ACTH. The various adrenal steroids that have synthesis controlled by CRH and ACTH as well as their metabolites may operate as neuroactive [14], neuroprotective [15] and immunoprotective [6] substances, affecting the somatic, psychosomatic and psychiatric health of patients [16,17,18,19].

Spa treatment can be an effective instrument for the reestablishment of physical and mood balance in patients with anxiety-depressive disorders. Our previous report on 70 female patients of a wide age range who were diagnosed with anxiety-depressive disorders demonstrated both positive changes in HPAA activity and improvements in three types of neurotic scores (and the summary score) based on a modified N-5 self-evaluation neurotic questionnaire [19] consisting of somatic, psychosomatic and psychiatric components [20,21]. Based on these results, the authors postulated a close association between the improvement of patients’ mood balance during the spa treatment and changes in the steroid metabolome of adrenal origin. To confirm this, 46 postmenopausal 50+ women with anxiety-depressive disorders who underwent the standardized one-month therapy with physical activity and an optimized daily regimen in a Czech spa were studied. Postmenopausal women are the most appropriate subjects for evaluating adrenal steroidogenesis, as the gonadal contribution does not substantially interfere in these patients [22]. Using a multivariate regression, this study investigated the relevance of these associations and attempted to interpret the results from the viewpoint of human physiology and pathophysiology. Particular attention was paid to neuroactive, neuroprotective and immunoprotective steroids of adrenal origin.

## 2. Results

### 2.1. Dependent Variables and Predictors

This study evaluated relationships between four dependent variables (scores for somatic, psychosomatic and psychiatric symptoms and the overall neurotic score) and 88 predictors using Spearman’s correlations, a multivariate regression with a reduction of dimensionality, the method of orthogonal predictions to latent structure (OPLS), and an ordinary multiple regression (MR). The predictors included the age of the patients and 88 steroids in the circulation. The steroid metabolome included the levels of C21 Δ^5^ steroids, C19 Δ^5^ steroids, C21 Δ^4^ steroids, C19 Δ^4^ steroids, estrogens, C21 and C19 5α/β-reduced steroids, 7α-hydroxy-, 16α-hydroxy, 7β-hydroxy and 7-oxo-derivatives of C19 Δ^5^ steroids, and 20α-dihydro-metabolites of C21 steroids (20α-dihydro-pregnanes).

The following circulating steroids were quantified by gas chromatography tandem mass spectrometry (GC-MS/MS) (*n* = 88) using our recently published method [23]: Pregnenolone, pregnenolone sulfate, 17-hydroxypregnenolone, 17-hydroxypregnenolone sulfate, 16α-hydroxypregnenolone, 20α-dihydropregnenolone, 20α-dihydropregnenolone sulfate, dehydroepiandrosterone (DHEA), DHEA sulfate (DHEAS), 7α-hydroxy-DHEA, 7-oxo-DHEA, 7β-hydroxy-DHEA, androstenediol, androstenediol sulfate, 5-androstene-3β,7α,17β-triol, 5-androstene-3β,7β,17β-triol, 5-androstene-3β,16α,17β-triol, 5-androstene-3β,16α,17β-triol sulfate, progesterone, 17-hydroxyprogesterone, 17α,20α-dihydroxy-4-pregnen-3-one, 16α-hydroxyprogesterone, 20α-dihydroprogesterone, conjugated 20α-dihydroprogesterone, androstenedione, testosterone, conjugated testosterone, 5α-dihydrotestosterone, estradiol, conjugated estradiol, 5α-dihydroprogesterone, allopregnanolone, allopregnanolone sulfate, isopregnanolone, isopregnanolone sulfate, pregnanolone, conjugated pregnanolone, epipregnanolone, conjugated epipregnanolone, 5α,20α-tetrahydroprogesterone, conjugated 5α,20α-tetrahydroprogesterone, 5α-pregnane-3α,20α-diol, conjugated 5α-pregnane-3α,20α-diol, 5α-pregnane-3β,20α-diol, conjugated 5α-pregnane-3β,20α-diol, 5β,20α-tetrahydroprogesterone, conjugated 5β,20α-tetrahydroprogesterone, 5β-pregnane-3α,20α-diol, conjugated 5β-pregnane-3α,20α-diol, 5β-pregnane-3β,20α-diol, conjugated 5β-pregnane-3β,20α-diol, 17-hydroxyallopregnanolone, 17-hydroxyallopregnanolone sulfate, 17-hydroxypregnanolone, 17-hydroxypregnanolone sulfate, 5α-pregnane-3α,17α,20α-triol, conjugated 5α-pregnane-3α,17α,20α-triol, 5α-pregnane-3β,17α,20α-triol, conjugated 5α-pregnane-3β,17α,20α-triol, 5β-pregnane-3α,17α-20α-triol, conjugated 5β-pregnane-3α,17α,20α-triol, 5α-androstane-3,17-dione, androsterone, androsterone sulfate, epiandrosterone, epiandrosterone sulfate, etiocholanolone, etiocholanolone sulfate, epietiocholanolone, epietiocholanolone sulfate, 5α-androstane-3α,17β-diol, conjugated 5α-androstane-3α,17β-diol, 5α-androstane-3β,17β-diol, conjugated 5α-androstane-3β,17β-diol, 5β-androstane-3α,17β-diol, conjugated 5β-androstane-3α,17β-diol, cortisol, cortisone, corticosterone, 5α,20α-tetrahydrocorticosterone, 5β,20α-tetrahydrocorticosterone, 11β-hydroxyandrostenedione, 11β-hydroxyandrosterone, 11β-hydroxyandrosterone sulfate, 11β-hydroxyepiandrosterone, 11β-hydroxyepiandrosterone sulfate, 11β-hydroxyetiocholanolone, 11β-hydroxyetiocholanolone sulfate.

### 2.2. Relevant Changes after Intervention with Physical Activity

The significant changes (*p* < 0.05) during the intervention with physical activity in predictors and dependent variables are summarized in Table 1. In the Δ^5^ steroidogenic pathway, an increasing trend was observed for pregnenolone and its sulfate, 17-hydroxypregnenolone and its sulfate, 16α-hydroxypregnenolone, 20α-dihydropregnenolone, DHEA, 7α-hydroxy-DHEA, and androstenediol. In the Δ^4^ steroidogenic pathway, a similar trend was found for 17-hydroxyprogesterone, 16α-hydroxyprogesterone, and androstenedione. The situation was similar for various 5α/β-reduced metabolites of the Δ^4^ steroids, such as isopregnanolone sulfate, conjugated epipregnanolone, 5α-pregnane-3α,20α-diol, 17-hydroxyallopregnanolone and its sulfated counterpart, 17-hydroxyallopregnanolone and conjugated 17-hydroxyallopregnanolone, conjugated 5α-pregnane-3α,17α,20α-triol, epiandrosterone, epietiocholanolone sulfate and conjugated 5β-androstane-3α,17β-diol. In addition, glucocorticoids such as cortisol and corticosterone increased after intervention, as did the 5α/β-reduced metabolites of corticosterone and 11β-hydroxyandrostenedione. The ratio cortisol/DHEA-S significantly increased after the treatment (Table 1).

All dependent variables, i.e., the somatic symptoms score, psychosomatic symptoms score, psychiatric symptoms score, and overall neurotic symptoms score, significantly decreased after the intervention, with the most pronounced decline in the psychosomatic symptoms score of −71.4 (−80, −50) % (showed as medians with quartiles) (Table 1).

### 2.3. Analysis of Relationships between Neurotic Symptoms and Predictors

#### 2.3.1. Somatic Symptoms

**Figure 1:**
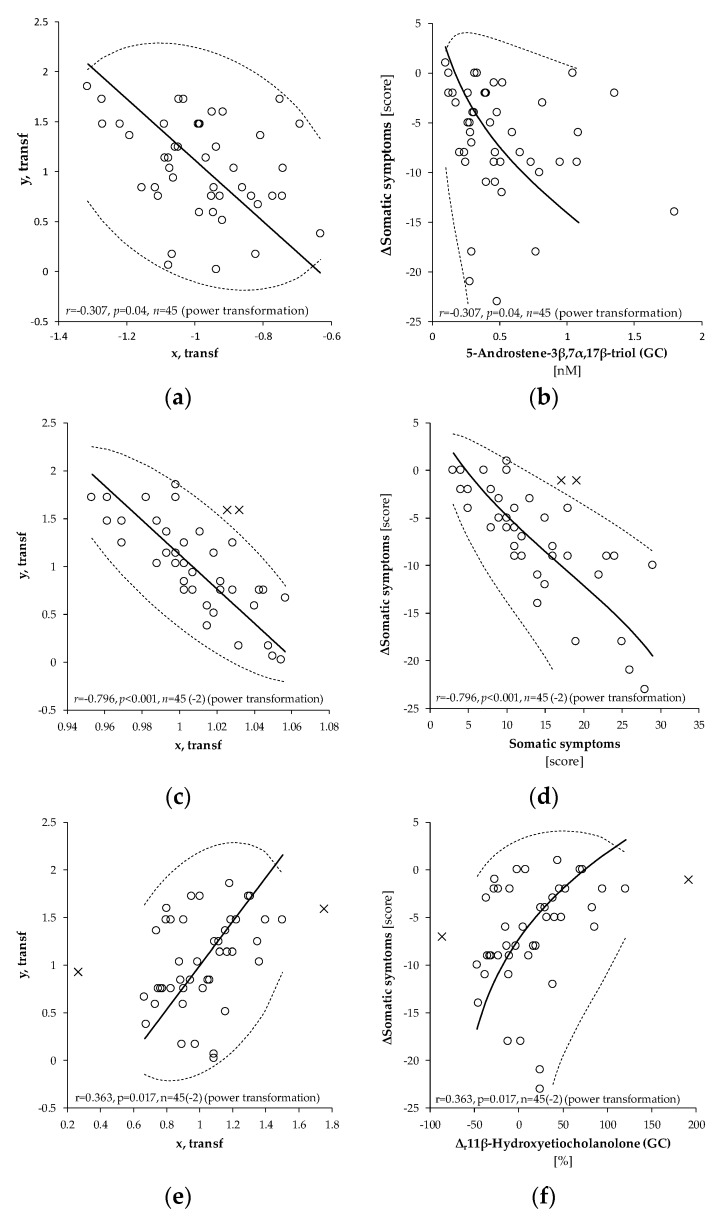
Pearson’s correlation between transformed values of absolute differences (after intervention-basal) of somatic symptoms score (y, transf; ΔSomatic symptoms) and transformed values of (**a**) 5-androstene-3β,7α,17β-triol (x, transf, 5-Androstene-3β,7α,17β-triol), (**c**) basal somatic symptoms (x, transf, somatic symptoms), and (**e**) of relative differences Δ_r_ ((value after intervention – basal value)/basal value) for 11β-hydroxyetiocholanolone (x, transf, Δ_r_11β-hydroxyetiocholanolone). Power transformations were used to attain Gaussian distribution and to stabilize the variance in each variable. The straight full line, dashed curve, and circles in panels (**a**,**c**,**e**) represent the principal axis, 95% confidence interval, and experimental points, respectively in transformed data. The corresponding curves and circles in (**b**,**d**,**f**) depict the same after re-transformation to the original scales. The symbols *r*, *p*, and *n* represent the Pearson’s correlation coefficient, its statistical significance and number of measurements, respectively. The number in parenthesis signifies the number outlying points outside of the 95% confidence ellipsoid that were excluded from the correlation analysis. These points are marked as crosses.

The relationships between the decline of somatic symptoms after intervention and the predictors as evaluated by OPLS and MR are shown in Table 2. The OPLS model showed that the decrease of somatic symptoms after intervention positively correlated with numerous C21 and C19 steroids at the beginning of the treatment (basal steroids) and with basal (before intervention) scores of somatic symptoms (Figure 1a,b). The decline of somatic symptoms also positively correlated with their basal values (Figure 1c,d). Alternatively, the decline of somatic symptoms negatively correlated with relative increases (Δ_r_ − calculated as (value after intervention − basal value)/basal value)) of 16α-hydroxypregnenolone, 11β-hydroxyandrostenedione and its unconjugated 5α/β-reduced-metabolites (Figure 1e,f). The predictors in the OPLS model explained 87.4% (76.2% after cross-validation) of the variability in the reduction of somatic symptoms scores. The MR analysis demonstrated that the positive associations between the decrease of somatic scores and levels of basal steroids in general and the basal scores of somatic symptoms were due to only four predictors: 5α-androstane-3,17-dione, the basal scores of somatic symptoms, and decreases of Δepietiocholanolone, and Δ11β-hydroxyandrosterone (Δ is calculated as value after intervention – basal value). These predictors were specific and did not share the variability with other predictors and remained significant in the MR.

#### 2.3.2. Psychosomatic Symptoms

The associations between the reduction of psychosomatic symptoms after intervention and the predictors are shown in Table 3. As in the case of somatic symptoms, the OPLS model showed that the decline in the psychosomatic symptoms score after intervention was positively correlated with numerous C21 and C19 steroids at the beginning of the treatment (basal steroids) and with the psychosomatic symptoms before intervention. Only a single negative correlation with an absolute reduction of psychosomatic symptoms was found for Δ11β-hydroxyandrostenedione. The predictors in the OPLS model explained 84.7% (72.9% after cross-validation) of the variability in the reduction of psychosomatic symptoms scores. In the MR analysis, the situation was similar to somatic symptoms. The decline of psychosomatic symptoms showed specific positive relationships with basal values of basal psychosomatic symptoms and with basal levels of conjugated 5β,20α-tetrahydroprogesterone (GC) and 17-hydroxyallopregnanolone sulfate and a negative specific positive association with Δ11β-hydroxyandrostenedione (GC).

#### 2.3.3. Psychiatric Symptoms

The relationships between the decline of psychiatric symptoms after intervention and the predictors are shown in Table 4. The OPLS model showed that the reduction of psychiatric symptoms scores after intervention positively correlated with psychiatric symptoms before intervention, but not with basal steroids. Instead, several negative correlations with absolute reductions of psychiatric symptoms were found with the Δ_r_ of steroids. The predictors in the OPLS model explained 43.5% (38.6% after cross-validation) of the variability in the decrease of psychiatric symptoms scores. The MR analysis for the decline of psychiatric symptoms showed specific relationships with all relevant predictors.

#### 2.3.4. Overall Neurotic Symptoms

The relationships between the decline of overall neurotic symptoms after intervention and the predictors as evaluated by OPLS and MR are shown in Table 5. The OPLS model showed that the decrease of somatic symptoms scores after intervention positively correlated with a variety of C21 and C19 steroids at the beginning of the treatment (basal steroids) and with overall neurotic symptoms before intervention. Alternatively, there were negative correlations of an absolute reduction of overall neurotic symptoms with Δ_r_ of 16α-hydroxypregnenolone, 11β-hydroxyandrostenedione and its unconjugated 5α/β-reduced-metabolites. The predictors in the OPLS model explained 65.5% (31% after cross-validation) of the variability in the reduction of overall neurotic symptoms. Apart from the basal overall neurotic symptoms, the predictors of the decline in overall neurotic symptoms were almost all unspecific, as indicated by the absence of their significance in the MR model.

## 3. Discussion

To evaluate the effects of the spa treatment with physical activity on the HPAA and consequently on the steroidome in the circulation of 46 postmenopausal females aged 50+, this study primarily followed the basal steroid values and their relative changes after the treatment intervention. However, our previous study did not evaluate which predictors were associated with the observed improvements of physical and mental health, and therefore our previous study did not interpret the expected associations from physiological and pathophysiological viewpoints. Consequently, these aspects were the main focus of the present study. The results shown in Table 1, Table 2, Table 3, Table 4 and Table 5 demonstrate the relevance of numerous steroids on the effect of intervention and relationships between neurotic scores and the steroid metabolome. As displayed in Table 6, most of these steroids are bioactive.

### 3.1. Increased Adrenal Steroidogenesis after Intervention

The consistent increase in a variety of steroids after intervention points to the reinstatement of the HPAA during the treatment (Table 1). Furthermore, most of these steroids, or at least their precursors or catabolites, are desirably bioactive, neuroactive, neuroprotective, and immunoprotective substances (Table 6). Therefore, as discussed later in the text, their increase during the intervention was likely to have been largely beneficial for the patients.

### 3.2. Positive Relationships between Initial Values of Neurotic Symptoms and Their Decline after Intervention

The strong positive associations between the initial values of neurotic symptoms and their decline after intervention were found for all components and for the overall score of the N-5 neurotic questionnaire (Table 2, Table 3, Table 4 and Table 5). This means that the higher neurotic scores were at the beginning of the treatment, the more pronounced their decline were after the intervention (see Figure 1c,d). Naturally, if the neurotic scores were low at the beginning of the treatment, the treatment effect should have been less pronounced.

### 3.3. Positive Associations between the Initial Adrenal Activity and the Drop of Neurotic Scores after Intervention

The levels of numerous steroids at the beginning of the treatment showed positive associations with a decrease of somatic (Table 2) and psychosomatic (Table 3) components of the N-5 neurotic questionnaire and with a decrease of its overall score (Table 5). This means that the better adrenal functioning was at the beginning of the treatment, and the more pronounced the alleviation of somatic and psychosomatic symptoms were after the intervention (see Figure 1a,b). The positive associations between the initial values of neurotic symptoms and their decline after the intervention and, at the same time, the positive associations between the adrenal activity and the decline of neurotic symptoms after the intervention, indicate that even high levels of neurotic impairment may be alleviated during the treatment with physical activity, provided that the initial adrenal activity is not seriously disrupted.

### 3.4. Inverse Relationships between the Drop of Neurotic Scores and Increase in the Levels of 5α/β-Reduced C19 Steroids

This study also found negative relationships between the decrease of neurotic scores and relative increases (Δ_r_) of various 5α/β-reduced androgen metabolites, and 16α-hydroxypregnenolone (for psychiatric symptoms) (Table 2, Table 3, Table 4 and Table 5). The greater the increase of these substances, the smaller the decline in the somatic, psychosomatic, psychiatric components of the neurotic symptoms and overall neurotic scores after the intervention (see Figure 1e,f). The fall in the somatic symptoms after the intervention negatively correlated with 5α/β-reduced 17-oxo-steroids such as 5α-androstane-3,17-dione, androsterone, epiandrosterone, etiocholanolone, 11β-hydroxyandrosterone and 11β-hydroxyetiocholanolone (Table 2), which are relatively stable catabolites of adrenal steroids. In the psychosomatic symptoms, this relationship was significant only for Δ_r_ of 11β-hydroxyandrostenedione (Table 3). The decrease of psychiatric symptoms negatively correlated with various C19 and C21 steroids (Table 4), and the decrease in the overall neurotic score negatively correlated with C19 11β-hydroxy-metabolites and 16α-hydroxypregnenolone (Table 5). These findings might be associated with high levels of CRH, which is a stress marker and at the same time a direct stimulator of androgen synthesis in the ZR [4,5,8,12,13]. Therefore, the increased production of androgen metabolites during the treatment may indicate the persistence of stress, and consequently a less efficient effect of the intervention.

### 3.5. Psychiatric Component of the Neurotic N-5 Questionnaire and Activity of Adrenal Cortex

In contrast to somatic and psychosomatic components of the neurotic N-5 questionnaire (Table 2 and Table 3), the decline in the psychiatric component did not depend on the initial levels of steroids (Table 4). However, there were some steroids for which the relative increase (Δ_r_) positively or negatively correlated with the decline in psychiatric symptoms after the treatment. The group of positively correlated steroids included sulfates of androstenediol, androsterone, epiandrosterone, and conjugated 5α-androstane-3α,17β-diol, while the negatively correlated group consisted of 16α-hydroxypregnenolone, conjugated 5α,20α-tetrahydroprogesterone, conjugated 5β,20α-tetrahydroprogesterone, 11β-hydroxyandrostenedione, 11β-hydroxyandrosterone, 11β-hydroxyepiandrosterone, and 11β-hydroxyetiocholanolone. The higher the increase of 11-deoxy-androgens and the lower the increase of 11β-hydroxy-androgens, 5α,20α-tetrahydroprogesterone, conjugated 5β,20α-tetrahydroprogesterone, and 16α-hydroxypregnenolone, the more pronounced was the decrease in psychiatric components. Whereas the positively correlated group is metabolites of DHEA sulfate, which is formed in the ZR, the second group is more associated with the activity of the adrenal *zona fasciculata* (ZF). This is due to the C21 steroids being formed in the ZF, and the 11β-hydroxy-androgens being at least partly the products of corticoids after a conversion catalyzed by CYP17A1 [74]. The data thus indicates that the decline in psychiatric symptoms after the intervention may be associated with an increasing ratio of ZR activity to ZF activity.

### 3.6. The Effect of Treatment with Physical Activity on the Levels of Bioactive Steroids and Their Actions

#### 3.6.1. Anti-Glucocorticoid Immunoprotective Steroids

The most important finding from the viewpoint of patients’ welfare is that immunoprotective (stimulate immune response but suppress autoimmunity) Δ^5^ C19 steroids such as DHEA, androstenediol, 7α-hydroxy-DHEA, 7-oxo-DHEA, and 5-androstene-3β,7α,17β-triol [6,28,29,30,31,32] (see Table 6) showed a significant increase after intervention (Table 1). Furthermore, the initial levels of these steroids positively correlated with a decline of somatic and psychosomatic symptoms as did the overall scores of the N-5 neurotic questionnaire (Figure 1a,b, Table 2, Table 3 and Table 5). The Δ^5^ C19 steroids, and especially their 7α/β-7-oxo- and 16α-hydroxy-metabolites, are widely known as anti-glucocorticoid and immunoprotective substances [6,28,29,30]. These results demonstrate that at least the activity of the ZR is augmented after intervention, and that its activity at the beginning of the treatment predicts the improvement in somatic and psychosomatic components of the N-5 neurotic questionnaire. Apart from these bioactive substances, further adrenal C19 steroids and their 5α/β-reduced metabolites such as androstenedione, 5α-androstane-3,17-dione, androsterone, epietiocholanolone sulfate, conjugated 5β-androstane-3α,17β-diol, 5α-androstane-3α,17β-diol showed similar associations with a decline in somatic and psychosomatic symptoms after the intervention (Table 2 and Table 3). The levels of Δ^5^ C19 steroids are prominently age-dependent [75,76] and their decline during life is tightly associated with the shrinkage of cells in the ZR [77]. Therefore, the increase of circulating adrenal C19 steroids and their 5α/β-reduced metabolites after intervention with physical activity in females aged 50+ indicates that their *zona reticularis* (ZR) turns younger. Moreover, the success of the intervention depended on the initial state of the ZR. The younger the patient’s ZR at the beginning of the treatment, the more prominent the reduction of somatic and psychosomatic symptoms after its completion.

Apart from the peripheral production, DHEA may be locally synthesized in the brain. The expression of CYP17A1 catalyzing its formation was detected in the CNS. The brain DHEA synthesis with (reviewed by Manninger [78]) and without CYP17A1 catalysis [79] was reported. Asaba et al. reported that the efflux of DHEAS by transport proteins from the brain was approximately ten times more rapid than the efflux from the periphery into the brain [80]. However, in the earlier study, Wang et al. [81] demonstrated the transport of peripherally applied pregnenolone sulfate across the BBB, which was approximately ten times slower when compared to the unconjugated pregnenolone. A recent study by Qaiser et al. [82] also demonstrates an uptake of peripherally applied [^3^H]-DHEAS and [^3^H]-PregS, which is more rapid for the latter (less polar) conjugate. A consequent extensive (>50%) desulfation of both conjugates within 0.5 min of uptake was observed. No further metabolism was detected for the pregnenolone sulfate beyond the liberation of free steroid pregnenolone. Alternatively, the DHEAS underwent conversion to androstenediol in both the steroid sulfate and the free steroid fractions with some additional formation of androstenedione in the latter. These data also point to the minor contribution of the brain CYP17A1 enzyme on the DHEA/DHEAS brain concentrations in comparison with the impact of DHEAS transport from the periphery. In addition, the authors have previously found minor activity of steroid sulfatase SULTA1 but moderate sulfatase (STS activity) in the primate brain [83,84]. Significant correlations have also been reported between various steroids in the periphery and cerebrospinal fluid [85] at pronouncedly higher peripheral levels of steroids also indicating the substantial participation of the steroid transport from periphery on the steroid milieu in the brain. At the same time, the higher levels of steroids in the brain tissue in comparison with both the circulation and CSF (with greater differences for the non-polar steroids) could be explained by the fairly non-polar character of these tissues extracting the lipophilic substances. Moreover, the expression of CYP17A1 in the primate brain is negligible when compared to that in peripheral tissues [86,87]. The aforementioned data demonstrates the major contribution of circulating steroid sulfates as hormone precursors at the blood–brain barrier, with implications for various physiological and pathological processes, including ageing.

#### 3.6.2. Ergosteroids

The 7α-hydroxy and 7-oxosteroids also function as ergosteroids, activating the enzymes glycerol-3-phosphate dehydrogenase and malic enzyme and inducing increased heat production [31], the final effect of which may be comparable to thyroid hormones [31,32]. From the steroids included in this study, a thermogenic effect has also been exhibited by glucocorticoids [31] and unconjugated 5β-steroids such as pregnanolone, 5β-pregnane-3α,20α-diol, etiocholanolone, and 11β-hydroxyetiocholanolone [33] (Table 6). However, the thermogenic effects of these steroids are associated with a β-adrenoceptor (β-AR) pathway in the brown adipose tissue [88], in addition to the stimulation of the release of interleukin-1 and other pro-inflammatory cytokines from leukocytes [31].

#### 3.6.3. Glucocorticoids

Glucocorticoids are another group of bioactive steroids that are widely known as immunosuppressive and anti-inflammatory mediators and may also function as ergosteroids via the β-adrenoceptor (β-AR) pathway in the brown adipose tissue [88]. Basically, endogenous glucocorticoids (GC) activate both glucocorticoid (GR) and mineralocorticoid receptors (MR) in the central nervous system (CNS) regulating the stress response via rapid non-genomic MR-mediated and slower genomic GR-mediated effects. The MR bind cortisol and corticosterone has approximately 10-fold higher affinity when compared to GR. Whereas the levels of circulating aldosterone are by 1-2 orders of magnitude lower (depending on diurnal changes and stress events), when compared to the endogenous glucocorticoids (cortisol and corticosterone). The latter steroids are the primary ligands for both MR and GR in the CNS [89]. Furthermore, besides the adrenal androgens, the aldosterone secretion and renin activity also decline with age [90], which further support the major role of glucocorticoids during aging.

The MR are primarily expressed in limbic neurons of the hippocampus (HC), lateral septum and amygdala and bind to various steroids, including the mineralocorticoids (aldosterone and 11-deoxycorticosterone), glucocorticoids (cortisol and corticosterone), and progesterone [89]. While the rapid MR-mediated response is targeted to emotions and instant coping decisions, the slow GR-mediated response is directed to contextualization, rationalization and memory storage of the stress experience. As the GR antagonists induce an impairment of memory storage, the MR antagonists inhibit the memory retrieval [89]. The hippocampus (HC) and prefrontal cortex (PFC) primarily inhibit the limbic-HPAA activity, while amygdala activates the stress response [90]. While the MR regulates a tonic effect of glucocorticoids in the brain at basal levels, the activation of the GR blunts further activity of the stress response [89,90]. The amygdala response rises shortly after stress. After 1 h, the activity in the PFC and HC escalate as well, at a concurrent decline of amygdala actions. However, the very intense stress situations induce a delayed, more pronounced and long-lasting amygdala activity [89]. Whereas the activation of the MR promotes the hippocampal plasticity, GR exerts the opposite effect [90]. The MR principally control the threshold of reactivity of the HPAA during stress, which is generally considered to be a healthy state [89]. The balance between the MR and GR-mediated stages is associated with U-shaped dependence on the glucocorticoid concentration. In areas where the MR is expressed at a much lower level than GR, the glucocorticoid effects linearly depend on the hormone concentration [89]. An imbalance between MR-mediated rapid stage and GR-mediated slow stage of the stress response caused by changed functionality of one of the corticosteroid receptors, results in compromised stress responses and may increase the susceptibility to various diseases [89].

Aging is associated with weakened negative feedback on the cortisol secretion, due to impaired sensitivity of the HPAA [90]. The elevation of cortisol levels is frequently related to a lower cognitive status, anxiety, depression, dementia, and neurodegenerative diseases. Furthermore, the risk of developing insulin resistance increases with elevated cortisol levels in older people and the flatter diurnal slope of cortisol profile in seniors is associated with type 2 diabetes [90].

In contrast to adrenal androgens and sex steroids declining during aging, the data concerning mean cortisol concentrations are contradictory [90]. Nevertheless, numerous studies report that in seniors, cortisol shows a flattened circadian profile, with elevation at night [89] and attenuation of earlier morning level peaks [89,90,91]. The relationships between the cortisol secretion on one side and age, sex and body mass index on the other side was thoroughly evaluated in the recent study of Roelfsema et al. in 143 healthy adults spanning 7 decades and with a 2-fold body mass index (BMI) range [91]. The authors in that study found no sex differences in the mean 24-h cortisol concentrations in subjects older than 50 years. The aging increased the mean cortisol by 10 nmol/L per decade during the quiescent secretory phase and advanced the acrophase of the diurnal rhythm by 24 min per decade. Nevertheless, the total 24-h cortisol secretion rates as estimated by deconvolution analysis did not depend on age, sex and BMI. On the other hand, age and sex jointly determined the 24-h cortisol secretory profile (sex effects were restricted to age <50 years). The age effects elevated concentrations in the late evening and early night and advanced the timing of the peak diurnal rhythm.

Type 1 11β-hydroxysteroid dehydrogenase (HSD11B1), converting inactive 11-oxo-corticoids to their bioactive 11β-hydroxy-counterparts is expressed in the CNS and exhibits increased activity with aging. Alternatively, lower diurnal cortisol levels are associated with longevity [90]. Further, the HSD11B isoform, type 2 11β-hydroxysteroid dehydrogenase (HSD11B2) exerting the opposite effect is subtly distributed in the brain [89].

Our present data shows that the initial levels of GC do not directly correlate with neurotic symptoms, in contrast to their 11β-hydroxy-C19 catabolites such as 11β-hydroxyandrostenedione, 11β-hydroxyandrosterone sulfate, and 11β-hydroxyetiocholanolone, which may be formed either from glucocorticoids in a lyase step catalyzed by CYP17A1 or via 11β-hydroxylation of androgens [74] (Table 2 and Table 5). The initial levels of the aforementioned steroids positively correlated with the decline of somatic symptoms scores after the intervention (Table 2) and with the reduction of overall neurotic scores (Table 5). These data indicate that the functioning of corresponding enzymes such as CYP11B1, CYP17A1 and the balance between cortisol forming HSD1B1 and cortisol catabolizing HSD1B2 may be of importance for the effect of the spa treatment. 

A potential ambiguity was the slight but significant increase of glucocorticoids such as cortisol, corticosterone, and a more pronounced increase of their 3α,5α/β reduced corticosterone metabolites and increased cortisol/DHEA ratio after the treatment (Table 1). The response of DHEA to ACTH challenge pronouncedly weakens with advancing age due to shrinkage of steroid producing cell in adrenal ZR [9,90]. The explicit decline in the synthesis of adrenal androgens during aging at subtle changes in glucocorticoid levels resulting in obvious increase of the cortisol/DHEAS ratio during aging, may also contribute to the pathophysiology of age-related neurodegenerative diseases [90]. On the one hand, the increased cortisol levels may increase the risk of depression, but on the other hand, the cortisol diurnal rhythm is frequently disturbed in patients with psychiatric disorders, and a mild increase of cortisol levels may be a sign of restoration [92,93,94]. Furthermore, some data indicate an improved physical and cognitive performance in aging population during higher activity of the HPAA, compared with reduced activity of the axis [90]. In addition, our patients were treated with physical activity, which attenuates the HPAA response to psychological stress in aging female [95]. Finally, it should be pointed out that this study did not compare the whole diurnal profile in any stage of the treatment. The serum samples from our patients were collected early in the morning (at 7 a.m.) around the acrophase of the cortisol diurnal profile when the aging subjects exhibit lower cortisol levels than the younger ones [91]. Nevertheless, the remaining hormonal and mood changes indicating the restoration of adrenal activity after the treatment and the recent data of Roelfsema et. al. [91], allow speculation about a restoration of the cortisol diurnal profile towards the situation typical for the younger subjects i.e., to the lowering of cortisol levels in the night but their increase around the acrophase early in the morning. Therefore, the increase of morning cortisol levels may not stand for the shift of the whole diurnal profile to the higher values. Quite the reverse, cortisol morning elevation points to a positive alteration of its diurnal rhythm (including the observed cortisol elevation around the acrophase). The relationships between the performance of the brain MR and the shape of the cortisol diurnal profile also allow contemplation of at least partial restoration of the balance between MR and GR functioning in the CNS after the spa treatment.

#### 3.6.4. Progestogens

Apart from the key role of progestogens in human reproduction, these steroids are known as efficient neuroprotective substances operating in both the CNS and periphery and easily penetrating through the blood-brain barrier [96]. Even though progestogen levels are low and their effect is limited in postmenopausal women, this study recorded an increase of 17-hydroxyprogesterone and 16α-hydroxyprogesterone after the intervention (Table 1) as well as positive correlations of initial 20α-dihydroprogesterone with a decline of somatic symptoms (Table 2).

#### 3.6.5. Glutamatergic Steroids Modulating NMDA and AMPA/Kainate Receptors (Enhancement of Mental Activity, Protection from Excitotoxicity)

The sulfates of pregnenolone and 17-hydroxypregnenolone showed a significant increase after intervention, and initial allopregnanolone sulfate positively correlated with a decrease of psychosomatic symptoms, as did conjugated pregnanolone. The sulfates of pregnenolone, 17-hydroxypregnenolone and allopregnanolone are neuroexcitatory substances activating some subtypes of *N*-methyl-d-aspartate receptors (NMDAR) (Table 6) and enhancing mental activity via the stimulation of calcium influx into neurons, while conjugated pregnanolone is inhibitory [36,37,97]. At the same time, pregnenolone sulfate as well as conjugated pregnanolone are inhibitory and neuroprotective for another type of glutamate receptors, the AMPA/kainate (AMPAR/KAR) receptors [98] (Table 6). AMPAR/KAR receptor overactivation and/or changes in receptor subunits may induce excitotoxic neuronal damage or death [99] in various neurological diseases [100,101]. As previously demonstrated by Wang et al. [81], the influx of peripheral pregnenolone sulfate across the blood-brain barrier in a rat model was evident. This is of particular importance in humans, where the levels of peripheral pregnenolone sulfate range from several tenths to hundreds of nanomoles in non-pregnant subjects and even to concentrations of a tenth of a micromole in fetuses. In addition, the circulating conjugated pregnanolone at the beginning of the treatment positively corelated with the decline of somatic and psychosomatic symptoms and overall neurotic scores (Table 2, Table 3 and Table 5). To summarize, the relevant glutamatergic steroids showed positive changes after the intervention towards the enhancement of mental activity via the stimulation of NMDAR and a concurrent increase of neuroprotection via the inhibition of AMPAR/KAR, and the initial levels of some of them predicted the decline in somatic and psychosomatic symptoms after the intervention.

#### 3.6.6. GABAergic Steroids (Neural Inhibition and Protection, Analgesic Effects)

There were also some GABAergic steroids (Table 6) showing significant changes or relationships with components of the neurotic N-5 questionnaire. From the inhibitory positive modulators of type A γ-aminobutyric acid receptors (GABA_A_R), this study found an increase of 5α-pregnane-3α,20α-diol a (weak modulator) after the treatment, a positive correlation of initial androsterone levels (a weak modulator) with the decline of psychosomatic symptoms (Table 3), a positive correlation of initial 5β-pregnane-3α,20α-diol levels (weak modulator) with the decline of somatic symptoms (Table 2) and negative correlations of a relative increase of androsterone and etiocholanolone (a weak modulator) levels with the decline of somatic symptoms after the intervention (Table 2). From the excitatory negative modulators of the GABA_A_R, this study observed an increase in sulfates of isopregnanolone, which are relatively strong GABA_A_R modulators, and epiandrosterone (Table 1) as well as positive correlations of initial levels of the former steroid with the decline in psychosomatic symptoms (Table 3). These results point to a lower relevance of positive GABA_A_R modulators on the one hand, but to an additional excitatory effect of the negative GABA_A_R modulators on the influence of the positive NMDAR modulators on the other hand.

#### 3.6.7. Glycinergic Steroids (Regulation of Neuronal Activity and Pain Perception)

Glycine receptors (GlyR) occurring in higher brain regions and regulating the chloride influx into the neuronal cells, similarly as GABA_A_R, play a critical role in motor and sensory functions and participate in the pathophysiology of psychiatric disorders in which the mesolimbic dopaminergic system is involved, such as psychosis and drug addiction [102]. The GlyR are also involved in the pain pathways [103]. Apart from the excitatory effects on NMDAR, pregnenolone sulfate, showing an increase after intervention (Table 1), is also excitatory on the GlyR. Alternatively, unconjugated androsterone (the initial levels of which positively correlated with a decline in psychosomatic symptoms) and unconjugated epiandrosterone (showing a significant increase after intervention) function as inhibitory, positive glycinergic modulators (Table 6). Therefore, the impact of glycinergic steroids on the results of the intervention may be ambiguous.

#### 3.6.8. Steroids Negatively Modulating L-Type Voltage Gated Calcium Channels (Antihypertensive and Analgesic Effects)

L-type calcium voltage gated calcium channels (VGCC) operate in excitation-contraction coupling of skeletal, smooth, and cardiac muscles, and participate in the regulation of aldosterone secretion in the adrenal zona glomerulosa (ZG). The dysregulation of ZG results in various pathologies, such as hypertension and cardiological problems [104]. Therefore, steroid negative modulators of the L-type VGCC may be anti-hypertensive and cardioprotective. The levels of negative L-type VGCC modulators pregnenolone, its sulfate and conjugated epipregnanolone significantly increased after intervention, which may have been beneficial for the patients, but the levels of 20α-dihydropregnenolone, operating as a negative modulator, showed a slight decline (Table 1). Taken together, the overall effect of the steroid modulators regulating the permeability of L-type VGCC on the results of the intervention appears to be preferably antihypertensive and analgesic.

#### 3.6.9. Steroids Modulating T-Type Voltage Gated Calcium Channels (Anti-Nociceptive Effect)

As demonstrated on animal models with the application of various synthetic 5α/β-reduced steroids, these substances are anti-nociceptive, blocking pain perception on the peripheral level via the inhibition of T-type VGCC. The levels of numerous endogenous 5α/β-reduced steroids showed a significant increase after the intervention (Table 1), and there were positive relationships of their initial levels with somatic and psychosomatic symptoms and the overall neurotic score (Table 2, Table 3 and Table 5). Therefore, the increase of these substances during the intervention might have stimulated anti-nociceptive effects.

#### 3.6.10. Multiple Positive Effects of Pregnenolone and Pregnenolone Sulfate on Transient Receptor Potential Channels

Apart from neuroprotective actions on AMPA/KAR receptors, pregnenolone sulfate attenuates the permeability of ionotropic capsaicin/vaniloid TRPV1 receptors, which serve as a molecular gateway to the pain pathway [105]. Therefore, the significantly increasing levels this steroid conjugates after the intervention (Table 1) may have contributed to the increase of the anti-nociceptive effect instigated by the intervention. A further beneficial effect of increased pregnenolone sulfate after the intervention may be the inhibition of the excitatory non-selective cationic transient receptor potential TRPC5 channels [69], which participate in the pathophysiology of innate fear [106]. Moreover, pregnenolone sulfate and pregnenolone (both increasing after the intervention) activate insulin secretion from pancreatic β-cells via the stimulation of ionotropic melastatin TRPM3 receptors [70]. To summarize, all these data reflected multiple beneficial effects through the increase of pregnenolone and pregnenolone sulfate induced by the intervention.

#### 3.6.11. Steroids Affecting the Expression of Nuclear Pregnane X Receptors

From the steroid-dependent nuclear receptors potentially relevant to our data, the pregnane X receptors (PXRs) were also considered. The primary function of PXRs, which are sensitive not only to exogenous toxic substances but also to a number of endogenous steroids, is to induce a response upregulating the expression of proteins involved in the detoxification and clearance of these substances as well as various xenobiotics from the body [107]. The PXRs may be, for instance, a target for the inhibition of androgen activity [108], and PXR activation may also attenuate some inflammatory processes [109]. Numerous steroid PXR activators (Table 6) showed a consistent increase after the intervention (Table 1). This indicates that the consolidation of adrenal steroidogenesis after the intervention induced the improvement of mechanisms catabolizing and clearing various toxic xenobiotics, which would clearly contribute to the overall beneficial effect of the spa treatment.

## 4. Materials and Methods 

### 4.1. Patients

This study included 46 postmenopausal 50+ women (50-77 years of age) suffering from anxiety-depressive disorders (International classification of mental disorders; ICD 10 or MKN-10 F4.) who were in a stabilized state and underwent a standardized one-month therapy with physical activity and an optimized daily regimen in the Priessnitz spa in Jeseník (640 m a.s.l.), in the Moravian-Silesian Region of the Czech Republic. The median initial body mass index of the patients was 25.5 kg/m^2^ (quartiles 23.7, 31.2), which slightly but significantly declined by −1.2 (−2.68, 0) % (*p* < 0.001) during the therapy, and their median height was 1.64 m (quartiles 1.60, 1.70). The patients were on the same stable antipsychotic medication with serotonin reuptake inhibitors (SSRI) but the use of exogenous steroids was an exclusion criterion in the present study. The spa treatment included neither hydrotherapy nor drinking cures but only physical activities, as described elsewhere [110]. The samples and measurements were taken from all patients on the third day of the treatment and after the intervention.

### 4.2. Samples

The clinical protocol was approved by the Ethical Committee of the Institute of Endocrinology in 5 June 2017, with project identification code MHCZ-DRO (Institute of Endocrinology—EÚ, 00023761). Informed written consent was obtained from all participants. On the third day of the intervention (after overcoming the initial stress from the different environment and diet) at 7 a.m., 10 mL of blood was drawn from the cubital or forearm vein in the morning (following overnight fasting) into a cooled plastic tube containing 100 μL of 5% EDTA. Plasma was obtained by centrifugation for 5 min at 2000× *g* at 4 °C, separated and frozen within half an hour of being drawn from the subject, and stored at −20 °C until analyzed. The second blood sampling was performed in the same way on the last day of the intervention. The patients were instructed not to eat nuts, bananas, or tomatoes, and not to drink coffee or black tea for two days before sampling.

### 4.3. Neurotic Scores

A modified self-evaluation neurotic N-5 questionnaire (Knobloch, Hausner) [20,21] was used to measure neurotic tendencies. This method is used worldwide in selecting and differentiating people with neurotic traits. The questionnaire consists of 33 items that represent basic neurotic symptoms:

1. Sleep disturbance; 2. daytime sleepiness; 3. fatigue and exhaustion; 4. low productivity; 5. headache; 6. perspiration; 7. vertigo; 8. faint feeling; 9. vomiting and nausea after anger; 10. hot and cold flashes; 11. shivers, inner need; 12. inattention and lack of concentration; 13. irritability and short temper; 14. memory retention; 15. backbone, muscle, and joint pain; 16. heart palpitations, heart pain; 17. breathing problems; 18. absence of appetite; 19. diarrhea or constipation; 20. blushing or turning pale; 21. pessimism; 22. sentimentality or hypersensitivity; 23. sorrowfulness or bad mood; 24. “I have no desire to do anything”; 25. indefinite anxiety or tension feeling; 26. worry about health or fear about life; 27. groundless fear lined to certain situation on the street, in crowd, on the bridge etc. ; 28. excessive fear from future; 29. self-doubt; 30. diffidence; 31. unpleasant feeling as in a dream, or feelings of non-existence; 32. intrusive thoughts or compulsion to action; 33. unpleasant feelings of stupor.

The intensity of the symptoms was self-estimated by patients using a 4-level scale: 0 = no, 1 = mild, 2 = high, 3 = extreme. The total score (from the viewpoint of neurotic tendency) was interpreted as follows: Normal (0–11), Mildly neurotic (12–22) Highly neurotic (23–33), Extremely neurotic (>34). The somatic and psychosomatic disturbances were reflected in questions 1–11 and 12–22, respectively, while psychiatric symptoms were represented by questions 23–33.

### 4.4. Analytical Methods

The GC-MS/MS steroid quantification method was described in detail in our recent article [23].

### 4.5. Terminology of Steroid Polar Conjugates

Concerning the terminology of the steroid polar conjugates used here, the term steroid sulfate was used in the case of the dominance of 3α/β-monosulfate over other steroid conjugate forms, while the term conjugated steroid was used in the case of comparable amounts of conjugate forms (sulfates, disulfates, and glucuronides). This terminology was based on the relevant literature, with appropriate citations for each steroid as follows: Preg sulfate [111,112], 20α-dihydropregnenolone sulfate, dehydroepiandrosterone (DHEA) sulfate [112,113,114], 5-Adiol sulfate [112,113], allopregnanolone sulfate, isopregnanolone sulfate [115], conjugated pregnanolone (sulfate + glucuronide) [116]), conjugated 5α-pregnane-3β,20α-diol (3β,20α-disulfate + 3β-sulfate) [116], conjugated 5β-pregnane-3α,20α-diol (3β,20α-disulfate + 3β-sulfate + glucuronide) [116], androsterone sulfate [112,113], epiandrosterone sulfate [112,113], etiocholanolone sulfate [117], epietiocholanolone sulfate, conjugated 5α-androstane-3α,17β-diol (glucuronide + sulfate [113], and conjugated 5α-androstane-3β,17β-diol (sulfate + glucuronide [113]).

### 4.6. Statistical Analysis

The absolute differences (Δ) of changes during spa treatment (calculated as the level at end of treatment—the level at the beginning of the spa treatment) as well as the relative (Δ_r_) changes (calculated as the ratio of absolute values to initial values in per cent) were evaluated by a robust Wilcoxon’s paired test using NCSS 12 statistical software from Number Cruncher Statistical System (Kaysville, UT, USA).

The relationships between changes of individual scores for somatic, psychosomatic, and psychiatric symptoms and total neurotic score on the one hand and steroid levels and further predictors and their relative changes on the other hand, were evaluated using a multivariate regression with a reduction of dimensionality (the method of orthogonal projections to latent structures, OPLS) to estimate the relevance and statistical significance of predictors. An ordinary multiple regression (MR) was employed for assessments of the specific contribution (uncorrelated with other predictors) for individual predictors [118,119,120]. The OPLS is effective in coping with the problem of severe multicollinearity within a matrix of predictors (high intercorrelations) where the MR fails to correctly evaluate such data. It is clear that the predictors in our data set (steroids in metabolic pathways) are highly intercorrelated.

Due to the non-Gaussian data distribution and the non-constant variance in most steroids, the original continuous variables for OPLS analysis were transformed by power transformations prior to further processing to attain data symmetry and homoscedasticity [121]. The statistical software Statgraphics Centurion 18 from Statpoint (The Plains, VA, USA) was used for the power transformations.

Pearson’s correlations were also used to evaluate the relationships between data transformed by power transformations. The two-dimensional non-homogeneities were detected using Hotelling’s T2 statistics and then excluded. NCSS 12 statistical software was used for the correlation analysis.

## 5. Conclusions

This study demonstrates that the specialized spa therapy with physical activity and an optimized daily regimen substantially improved the endocrine balance in postmenopausal females, aged 50+, resulting in a persisting consolidation of their mood and physical health. The consistent increase of adrenal steroidogenesis after intervention points to a reinstatement of hypothalamic-pituitary-adrenal axis during the treatment. The steroids, showing an increase after the intervention, are mostly bioactive. The increases of many steroids were likely beneficial to patients, including immunoprotective adrenal androgens and their metabolites, neuroactive steroids predominantly stimulating mental activity and at the same time protecting from excitotoxicity, steroids suppressing pain perception and fear and consolidating insulin secretion, as well as steroids improving the catabolism and clearance of various toxic xenobiotics via the activation of mechanisms dependent on pregnane X receptors. As expected, the higher the neurotic scores at the beginning of the treatment, the more pronounced the decline after the intervention. The positive associations between the initial values of neurotic symptoms and their decline after the intervention and, at the same time, the positive associations between initial adrenal activity and the decline of neurotic symptoms after the intervention, indicate that even high-level neurotic impairments may be alleviated by such therapy provided that the initial adrenal activity is not seriously disrupted. Our data also indicate that the decline of psychiatric symptoms after the intervention may be associated with an increasing ratio of ZR to ZF activities. Nevertheless, more detailed studies are required to confirm our findings and to elucidate the corresponding mechanisms of actions.

## Figures and Tables

**Table 1:** The relative *^a^* changes of steroids, further predictors and neurotic scores and absolute *^b^* changes of neurotic scores after intervention with physical activity (displayed as medians with quartiles). Only significant changes (*p* < 0.05) are shown.

**Variable**	**Basal Values**	**Δ_r_ [%] *^a^***	***p*-Value *^c^***
Age [years]	58 (55, 61)	---	---
Pregnenolone [nM]	0.935 (0.717, 1.18)	27 (−10.5, 58)	<0.001
Pregnenolone sulfate [nM]	79.6 (53.1, 106)	10.8 (−7.23, 25.4)	0.011
17-Hydroxypregnenolone [nM]	1.62 (1.09, 3.02)	52.4 (−4.44, 151)	<0.001
17-Hydroxypregnenolone sulfate [nM]	3.21 (2.18, 5.68)	41.2 (−9.57, 78.4)	<0.001
16α-Hydroxypregnenolone [nM]	0.31 (0.191, 0.493)	28.8 (−14.6, 88.5)	0.003
20α-Dihydropregnenolone [nM]	1.3 (0.949, 1.93)	10.6 (−11.8, 21.5)	0.038
Dehydroepiandrosterone (DHEA) [nM]	6.75 (4.3, 8)	24.4 (−3.65, 66)	<0.001
7α-Hydroxy-DHEA [nM]	1.2 (0.841, 1.92)	22.7 (−20.2, 53.7)	0.003
Androstenediol [nM]	1.29 (1.02, 1.97)	11.1 (−3.87, 34.2)	0.005
17-Hydroxyprogesterone [nM]	0.77 (0.502, 1.22)	43.1 (−23.5, 110)	0.001
16α-Hydroxyprogesterone [nM]	0.458 (0.287, 1.01)	32.7 (−25.6, 195)	0.002
Androstenedione [nM]	3.11 (1.98, 4.16)	25.6 (−12.6, 76.4)	0.003
Isopregnanolone sulfate [nM]	6.04 (4.52, 8.59)	5.58 (−9.3, 28)	0.031
Conjugated epipregnanolone [nM]	1.56 (0.837, 2.43)	8.84 (−11.6, 40.3)	0.013
5α-Pregnane-3α,20α-diol [nM]	0.0574 (0.0278, 0.114)	24.3 (−41, 87.6)	0.034
17-Hydroxyallopregnanolone [nM]	0.00813 (0.00426, 0.018)	30.5 (−55.1, 183)	0.046
17-Hydroxyallopregnanolone sulfate [nM]	0.928 (0.638, 1.53)	17.6 (−10.7, 40.2)	0.032
17-Hydroxypregnanolone [nM]	0.0365 (0.0132, 0.0743)	7.65 (−62.3, 135)	0.035
17-Hydroxypregnanolone sulfate [nM]	6.69 (4.56, 9.11)	18.5 (−7.25, 37.2)	0.022
Conjugated 5α-pregnane-3α,17α,20α-triol [nM]	388 (201, 740)	198 (40.2, 416)	<0.001
Epiandrosterone [nM]	1.18 (0.838, 1.52)	21.9 (−16.2, 43.7)	0.006
Epietiocholanolone sulfate [nM]	43.6 (23.5, 65.3)	19.6 (−1.59, 35.5)	<0.001
Conjugated 5β-androstane-3α,17β-diol [nM]	0.721 (0.389, 1.14)	15.5 (−13.9, 55.8)	0.015
Cortisol [nM]	413 (291, 472)	24.2 (−5.81, 61.6)	0.004
Cortisol/DHEAS	0.183 (0.0928, 0.416)	20.2 (−12.8, 71.7)	0.008
Corticosterone [nM]	12.6 (6.72, 17.5)	21.9 (−13.7, 129)	<0.001
5α,20α-Tetrahydrocorticosterone [nM]	0.0848 (0.0442, 0.193)	50.8 (−37.8, 254)	0.001
5β,20α-Tetrahydrocorticosterone [nM]	0.433 (0.218, 0.84)	22.6 (−35.4, 111)	0.013
11β-Hydroxyandrostenedione [nM]	136 (84, 201)	12.9 (−12.6, 53.7)	0.008
Somatic symptoms score	11 (8, 17)	−50 (−72.7, −25)	<0.001
Psychosomatic symptoms score	15 (11, 23)	−71.4 (−80, −50)	<0.001
Psychiatric symptoms score	12.5 (5, 18)	−57.3 (−86, −36.1)	<0.001
Overall symptoms score	39 (28, 57)	−56.8 (−76, −42.1)	<0.001
**Absolute Changes of Neurotic Symptoms**	**Basal Values**	**Δ *^b^***	***p*-Value *^a^***
Somatic symptoms score	11 (8, 17)	−6 (−9, −2)	<0.001
Psychosomatic symptoms score	15 (11, 23)	−10 (−15, −6)	<0.001
Psychiatric symptoms score	12.5 (5, 18)	−6 (−10.3, −3)	<0.001
Overall symptoms score	39 (28, 57)	−21 (−33, −13)	<0.001

^a^ Δ_r_ represents the relative change calculated as (value after intervention − basal value)/basal value, ^b^ Δ represents the absolute change calculated as (value after intervention − basal value); ^c^ the significance of changes was evaluated using Wilcoxon’s robust paired test.

**Table 2:** The relationships between the decline of somatic symptoms after treatment and predictors for the predictive component as evaluated by the orthogonal predictions to latent structure (OPLS) model and multiple regression (for details see Statistical analysis).

Data Type	Variable	OPLS (Predictive Component)				
		Component Loading	t-Statistic	R *^c^*	Regression Coefficient	t-Statistic
Relevant predictors (matrix X)	7α-Hydroxy-DHEA	0.168	2.80	0.302 *	−0.051	−0.52
	7-oxo-DHEA	0.117	1.70	0.210	−0.011	−0.18
	5-Androstene-3β,7α,17β-triol	0.215	3.64	0.385 **	0.163	1.84
	20α-Dihydroprogesterone	0.223	6.62	0.400 **	0.125	1.87
	Conjugated pregnanolone	0.241	4.00	0.432 **	0.080	0.77
	Conjugated 5β,20α-tetrahydroprogesterone	0.239	5.43	0.429 **	0.094	1.25
	5β-Pregnane-3α,20α-diol	0.130	3.36	0.233 **	−0.080	−0.88
	Conjugated 5β-pregnane-3α,20α-diol	0.198	3.49	0.356 **	−0.089	−1.22
	Conjugated 17-hydroxypregnanolone	0.265	5.20	0.476 **	0.120	1.10
	Conjugated 5β-pregnane-3α,17α,20α-triol	0.223	3.03	0.400 **	0.136	1.78
	5α-Androstane-3,17-dione	0.095	3.06	0.170 **	−0.148	−2.16 *
	5α-Androstane-3α,17β-diol	0.093	1.59	0.166	0.070	1.31
	11β-Hydroxyandrostenedione	0.281	4.81	0.505 **	0.057	0.89
	11β-Hydroxyandrosterone sulfate	0.197	2.36	0.353 *	0.095	0.77
	11β-Hydroxyetiocholanolone	0.280	3.33	0.502 **	0.032	0.34
	Somatic symptoms	0.476	6.13	0.855 **	0.704	15.75 **
	Δ_r_5α-Androstane-3,17-dione *^a^*	−0.090	−3.17	−0.162 **	−0.026	−0.22
	Δ_r_Androsterone	−0.028	−0.63	−0.051	0.107	0.88
	Δ_r_Epiandrosterone	−0.111	−1.65	−0.198	−0.010	−0.18
	Δ_r_Etiocholanolone	−0.197	−2.46	−0.353 *	−0.224	−1.65
	Δ_r_Epietiocholanolone	−0.066	−1.18	−0.119	0.144	2.48 *
	Δ_r_11β-Hydroxyandrosterone	−0.176	−2.93	−0.317 *	0.155	2.94 *
	Δ_r_11β-Hydroxyetiocholanolone	−0.203	−7.20	−0.364 **	−0.064	−0.99
(matrix Y)	ΔSomatic symptoms *^b^*	−1.000	−14.53	−0.935 **		
Explained variability of dependent variable		87.4% (76.2% after cross-validation)				

^a^ Δ_r_ represents the relative change calculated as (value after intervention − basal value)/basal value, ^b^ Δ represents the absolute change calculated as (value after intervention − basal value); ^c^ R represents component loadings expressed as correlation coefficients with a predictive component, * *p* < 0.05, ** *p* < 0.01.

**Table 3:** The relationships between the decline of psychosomatic symptoms after treatment and predictors for the predictive component as evaluated by the OPLS model and multiple regression (for details see Statistical analysis).

Data Type	Variable	OPLS (Predictive Component)				
		Component Loading	t-Statistic	R *^c^*	Regression Coefficient	t-Statistic
Relevant predictors (matrix X)	16α-Hydroxypregnenolone	0.208	2.88	0.341 *	−0.098	−1.35
	Dehydroepiandrosterone	0.205	2.28	0.337 *	−0.002	−0.02
	7α-Hydroxy-DHEA	0.194	2.24	0.318 *	−0.132	−1.01
	Androstenediol	0.225	2.68	0.368 *	0.023	0.24
	Androstenedione	0.252	3.20	0.412 **	0.086	1.15
	Allopregnanolone sulfate	0.192	8.04	0.314 **	0.028	0.33
	Isopregnanolone sulfate	0.189	3.74	0.309 **	−0.059	−1.08
	Conjugated pregnanolone	0.244	2.34	0.399 *	0.101	1.50
	Conjugated 5α-pregnane-3α,20α-diol	0.147	2.94	0.241 *	−0.047	−0.68
	Conjugated 5β,20α-tetrahydroprogesterone	0.219	3.23	0.359 **	0.157	3.19 **
	Conjugated 5β-pregnane-3α,20α-diol	0.191	2.50	0.312 *	−0.033	−0.56
	17-Hydroxyallopregnanolone sulfate	0.233	3.64	0.382 **	0.144	2.10 *
	Androsterone	0.188	3.40	0.309 **	0.027	0.67
	Epietiocholanolone sulfate	0.150	2.26	0.246 *	0.018	0.19
	Conjugated 5β-androstane-3α,17β-diol	0.128	1.34	0.210	−0.015	−0.19
	Psychosomatic symptoms	0.573	5.33	0.939 **	0.804	8.97 **
	Δ_r_11β-Hydroxyandrostenedione *^a^*	−0.269	−2.66	−0.440 *	−0.153	−1.91 *
(matrix Y)	ΔPsychosomatic symptoms *^b^*	−1.000	−9.07	−0.920 **		
Explained variability of dependent variable		84.7% (72.9% after cross-validation)				

^a^ Δ_r_ represents the relative change calculated as (value after intervention − basal value)/basal value, ^b^ Δ represents the absolute change calculated as (value after intervention − basal value); ^c^ R represents component loadings expressed as correlation coefficients with a predictive component, * *p* < 0.05, ** *p* < 0.01.

**Table 4:** The relationships between the decline of psychiatric symptoms after treatment and predictors for the predictive component as evaluated by the OPLS model and multiple regression (for details see Statistical analysis).

Data Type	Variable	OPLS (Predictive Component)				
		Component Loading	t-Statistics	R *^c^*	Regression Coefficient	t-Statistics
Relevant predictors (matrix X)	Psychiatric symptoms	0.355	5.91	0.658 **	0.195	3.42 **
	Δ_r_16α-Hydroxypregnenolone *^a^*	−0.288	−3.12	−0.535 **	−0.077	−2.22 *
	Δ_r_Conjugated androstenediol	0.301	3.38	0.559 **	0.086	2.38 *
	Δ_r_Conjugated 5α,20α-tetrahydroprogesterone	−0.192	−5.49	−0.355 **	−0.092	−2.43 *
	Δ_r_Conjugated 5β,20α-tetrahydroprogesterone	−0.238	−2.25	−0.442 *	−0.086	−2.74 *
	Δ_r_Androsterone sulfate	0.356	3.97	0.661 **	0.110	3.20 **
	Δ_r_Epiandrosterone sulfate	0.320	3.54	0.594 **	0.099	4.41 **
	Δ_r_Conjugated 5α-androstane-3α,17β-diol	0.296	3.99	0.549 **	0.068	2.77 *
	Δ_r_11β-Hydroxyandrostenedione	−0.234	−1.52	−0.434	−0.078	−2.06 *
	Δ_r_11β-Hydroxyandrosterone	−0.363	−5.97	−0.674 **	−0.085	−4.00 **
	Δ_r_11β-Hydroxyepiandrosterone	−0.148	−0.90	−0.274	−0.065	−2.69 *
	Δ_r_11β-Hydroxyetiocholanolone	−0.410	−9.39	−0.760 **	−0.123	−5.37 **
(matrix Y)	ΔPsychiatric symptoms *^b^*	−1.000	−5.12	−0.659 **		
Explained variability		43.5% (38.6% after cross-validation)				

^a^ Δ_r_ represents the relative change calculated as (value after intervention − basal value)/basal value, ^b^ Δ represents the absolute change calculated as (value after intervention − basal value); ^c^ R represents component loadings expressed as correlation coefficients with a predictive component, * *p* < 0.05, ** *p* < 0.01.

**Table 5:** The relationships between the decline of overall neurotic symptoms after treatment and predictors for the predictive component as evaluated by the OPLS model and multiple regression (for details see Statistical analysis).

Data Type	Variable	OPLS (Predictive Component)				
		Component Loading	t-Statistic	R *^c^*	Regression Coefficient	t-Statistic
Relevant predictors (matrix X)	16α-Hydroxypregnenolone	0.146	3.79	0.303 **	−0.160	−1.28
	Dehydroepiandrosterone	0.157	3.18	0.327 **	0.022	0.42
	7α-Hydroxy-DHEA	0.161	3.68	0.333 **	−0.124	−1.03
	Androstenediol	0.225	10.26	0.467 **	0.057	1.16
	5-Androstene-3β,7α,17β-triol	0.214	3.63	0.444 **	0.016	0.20
	Androstenedione	0.184	3.56	0.382 **	0.066	0.69
	Allopregnanolone sulfate	0.163	4.69	0.337 **	−0.045	−0.73
	Conjugated pregnanolone	0.242	3.72	0.502 **	0.066	0.64
	Conjugated 5β,20α-tetrahydroprogesterone	0.161	2.70	0.334 *	0.103	0.77
	5β-Pregnane-3α,20α-diol	0.136	2.46	0.282 *	−0.030	−0.31
	Conjugated 5β-pregnane-3α,20α-diol	0.198	4.94	0.410 **	−0.080	−1.20
	17-Hydroxyallopregnanolone sulfate	0.200	2.40	0.415 *	0.092	1.35
	Conjugated 17-hydroxypregnanolone	0.191	2.37	0.396 *	0.081	0.69
	5β-Pregnane-3α,17α,20α-triol	0.190	2.98	0.394 *	0.058	1.76
	Androsterone	0.128	3.19	0.265 **	0.044	0.68
	Epietiocholanolone sulfate	0.230	3.10	0.477 **	0.116	1.03
	5α-Androstane-3α,17β-diol	0.159	2.78	0.330 *	0.057	1.00
	5β-Androstane-3β,17β-diol	0.277	4.32	0.575 **	0.152	1.82
	11β-Hydroxyandrostenedione	0.191	2.99	0.397 *	0.028	0.28
	11β-Hydroxyetiocholanolone	0.197	2.52	0.409 *	0.103	0.88
	Overall neurotic symptoms	0.401	3.72	0.833 **	0.589	3.56 **
	Δ_r_16α-Hydroxypregnenolone *^a^*	−0.142	−2.69	−0.295 *	−0.007	−0.07
	Δ_r_11β-Hydroxyandrostenedione	−0.133	−2.54	−0.277 *	0.050	1.45
	Δ_r_11β-Hydroxyandrosterone	−0.172	−2.89	−0.356 *	0.107	1.14
	Δ_r_11β-Hydroxyepiandrosterone	−0.125	−2.20	−0.260 *	−0.017	−0.29
	Δ_r_11β-Hydroxyetiocholanolone	−0.187	−3.69	−0.387 **	−0.147	−1.62
(matrix Y)	ΔOverall neurotic symptoms *^b^*	−1.000	−7.22	−0.809 **		
Explained variability of dependent variable		65.5% (31% after cross-validation)				

^a^ Δ_r_ represents the relative change calculated as (value after intervention − basal value)/basal value, ^b^ Δ represents the absolute change calculated as (value after intervention − basal value); ^c^ R represents component loadings expressed as correlation coefficients with a predictive component, * *p* < 0.05, ** *p* < 0.01.

**Table 6:** List of bioactive steroids with significant effects of intervention and/or significant relationships with the decline of neurotic scores after 1-month of spa treatment with physical activity.

Steroid	Active Progestogens [24,25,26,27]	Active Glucocorticoids [25]	Immunoprotective Steroids [6,28,29,30,31]	Ergosteroids [31,32,33]	NMDAR Modulators [34,35,36,37,38,39,40,41,42,43]	AMPAR/KAR Modulators [41,44,45]	GABA_A_R Modulators [46,47,48,49,50,51,52,53,54,55]	GlyR Modulators [56,57,58]	L-type VGCCs Modulators [59,60,61,62,63,64]	T-type VGCCs Modulators [65,66]	TRPV1 Modulators [67,68]	TRPC5 Modulators [69]	TRPM3 Modulators [70,71,72]	PXR Modulators [73]
Pregnenolone					C+	C−	C−	C−	U−C−		C−	C−	(U+)C+	U+
20α-Dihydropregnenolone					C+		U−		U+					
17-Hydroxypregnenolone					C+									U+
Dehydroepiandrosterone			U+	U+	C+	C−	C−	C−			U−C−	C−	(U+)C+	U+C+
7α-Hydroxy-DHEA			U+	U+										
7-oxo-DHEA			U+	U+										
Androstenediol			U+	U+										
5-Androstene-3β,7α,17β-triol			U+	U+										
20α-Dihydroprogesterone	(U+)													
17-Hydroxyprogesterone	(U+)	(U+)												U+
16α-Hydroxyprogesterone	U+													
Androstenedione														U+
Allopregnanolone	U+				C+	U−	U+	U−	U+	?U−				U+
17-Hydroxyallopregnanolone										?U−				
Isopregnanolone							U−, C−			?U−				
Pregnanolone				U+	C−	C−	U+	U−	U+	?U−		(U−)C−		U+
Epipregnanolone							U−		C−	?U−			U+	
17-Hydroxypregnanolone										?U−				
17-Hydroxypregnanolone conjugated										?U−				
5α,20α-Tetrahydroprogesterone										?U−				
5α-Pregnane-3α,20α-diol							U+			?U−				U+C+
5α-Pregnane-3α,17α,20α-triol										?U−				
5β,20α-Tetrahydroprogesterone										?U−				
5β-Pregnane-3α,20α-diol				U+			U+			?U−				U+
5β-Pregnane-3α,17α,20α-triol										?U−				
5α-Androstane-3,17-dione										?U−				U+
Androsterone							U+C−	C−		?U−				U+
Epiandrosterone							C−	C−		?U−			C+	U+
Etiocholanolone				U+			U+			?U−	U−			U+
Epietiocholanolone										?U−				
5α-Androstane-3α,17β-diol							U+			?U−				U+
5β-Androstane-3α,17β-diol							U+			?U−				
Cortisol		U+		U+										
Corticosterone		U+		U+										U+
5α,20α-Tetrahydrocorticosterone										?U−				U+
5β,20α-Tetrahydrocorticosterone										?U−				
11β-Hydroxyetiocholanolone				U+						?U−				

U and C represent unconjugated and conjugated steroids, respectively and + and - represent the positive and negative modulation of receptors; ? indicates potential effect; () indicate minor effect; NMDAR = *N*-methyl-d-aspartate receptors, AMPAR = α-amino-3-hydroxy-5-methyl-4-isoxazolepropionic acid receptors; KAR = kainate receptors, GABA_A_R = type A γ-aminobutyric acid receptors; GlyR = glycine receptors; L-type VGCC = long-lasting voltage-dependent calcium channels; T-type calcium channels = transient opening calcium channels; TRPV1 = capsaicin receptor or transient receptor potential cation channel subfamily V member 1; TRPC5 = Short transient receptor potential channel 5; TRPM3 = transient receptor potential cation channel subfamily M member 3 channels.

## Abbreviations

50+	Older than 50 years of age
Δ	Difference, after treatment – before treatment
Δ_r_	Relative difference, (after treatment – before treatment)/before treatment
a.s.l.	Above sea level
ACTH	Adrenocorticotropic hormone, corticotropin
AKR1Cs	Subfamily 1C aldoketoreductases
AKR1D1	Steroid 5β-reductase
AMPA	α-amino-3-hydroxy-5-methyl-4-isoxazolepropionic acid receptors
CRH	Corticotropin releasing hormone, corticoliberin
CNS	Central nervous system
CYP3A4	Steroid 7β- and 16α-hydroxylating enzyme
CYP3A7	Steroid 7β- and 16α-hydroxylating enzyme
CYP11A1	Cholesterol desmolase
CYP17A1	Steroid C17-hydroxylase-C17,20-lyase
CYP19A1	Aromatase
CYP21A2	Steroid 21-hydroxylase
CYP7B1	Steroid 7α- and 16α-hydroxylating enzyme
CYP11B1	Steroid 11β-hydroxylase
CYP11B2	Aldosterone synthase
DHEA	Dehydroepiandrosterone
DHEAS	Dehydroepiandrosterone sulfate
GABA_A_R	Type A γ-aminobutyric acid receptors
GC-MS/MS	Gas chromatography tandem mass spectrometry
GlyR	Glycine receptors
HPAA	Hypothalamic-pituitary-adrenal axis
HSD3B1	Type 1 3β-hydroxysteroid dehydrogenase - Δ^5^/Δ^4^ isomerase
HSD3B1	Type 2 3β-hydroxysteroid dehydrogenase - Δ^5^/Δ^4^ isomerase
HSD11B1	Type 1 11β-hydroxysteroid dehydrogenase
HSD17Bs	17-Hydroxysteroid dehydrogenases
KAR	Kainate receptors
L-type VGCC	Long-lasting activation voltage-gated calcium channels
MR	Ordinary multiple regression
mRNA	Messenger ribonucleic acid
NMDAR	*N*-methyl-d-aspartate receptors
OPLS	Multivariate regression, method of orthogonal predictions to latent structure
PXR	Pregnane X receptors
SRD5As	Steroid 5α-reductases
STS	Steroid sulfotransferase
SULT2A1	Type 2A1 steroid sulfotransferase
T-type VGCC	Transient-opening voltage-gated calcium channels
TRPC5	Short transient receptor potential channel 5
TRPM3	Transient receptor potential cation channel subfamily M member 3, melastatin 3 receptors
TRPV1	Transient receptor potential cation channel subfamily V member 1, capsaicin receptors
ZF	Adrenal *zona fasciculata*
ZR	Adrenal *zona reticularis*
